# SOMATIC COMPLAINTS, A KEY FACTOR OF DEPRESSIVE SYMPTOMS ASSOCIATED WITH SUBJECTIVE MEMORY

**DOI:** 10.1093/geroni/igz038.2405

**Published:** 2019-11-08

**Authors:** Wonjeong Haavisto, Julie Blaskewicz Boron, Sherry Willis, Warner Schaie

**Affiliations:** 1 University of Nebraska, Omaha, Nebraska, United States; 2 University of Nebraska at Omaha, Omaha, Nebraska, United States; 3 University of Washington, Seattle, Washington, United States

## Abstract

Prior research found depressive symptoms and subjective memory to be associated with objective memory (OM) performance. One key factor of subjective memory, as measured by the Memory Functioning Questionnaire (MFQ), is General Frequency of Forgetting (GFF). However, few studies focused on identifying specific factors of depressive symptoms when examining associations between depressive symptoms, subjective memory and OM. Using structural equation modeling, cross-sectional associations of factors in the CES-D (depressive symptoms) to the MFQ (subjective memory) and OM were investigated in the Seattle Longitudinal Study (mean Age=72.39; SD=8.28; mean Education=15.12; SD=2.76; 58.4% female). Differential associations of the CES-D factors with the MFQ factors [n=389; RMSEA=.031; CFI=.973; TLI=.966] and the GFF subscales [n=389; RMSEA=.033; CFI=.971; TLI=.964] resulted. Only the CES-D somatic complaints factor was significantly associated with the GFF factor (β= -.45, p <.001), suggesting that people with more somatic complaints reported more memory concerns. The CES-D somatic complaints factor was negatively associated with the frequency of forgetting in daily life (β=-.36, p< .001) and forgetting while reading subscales (β= -.33, p < .001), indicating individuals that reported more somatic complaints experienced more frequent memory failures when performing daily activities and reading. Overall, a key CES-D factor, somatic complaints, emerged as influential to subjective memory, whereas there was no relation to OM. Further study of the longitudinal associations between the CES-D factors and subjective and objective memory is essential to determine the potential impact on memory deficits.

